# The integration of weighted gene association networks based on information entropy

**DOI:** 10.1371/journal.pone.0190029

**Published:** 2017-12-22

**Authors:** Fan Yang, Duzhi Wu, Limei Lin, Jian Yang, Tinghong Yang, Jing Zhao

**Affiliations:** 1 Department of Mathematics, Army Logistics University of PLA, Chongqing, China; 2 Rongzhi College of Chongqing Technology and Business, Chongqing, China; 3 School of Pharmacy, Second Military Medical University, Shanghai, China; 4 Institute of Interdisciplinary Complex Research, Shanghai University of Traditional Chinese Medicine, Shanghai, China; Universitatsmedizin Greifswald, GERMANY

## Abstract

Constructing genome scale weighted gene association networks (WGAN) from multiple data sources is one of research hot spots in systems biology. In this paper, we employ information entropy to describe the uncertain degree of gene-gene links and propose a strategy for data integration of weighted networks. We use this method to integrate four existing human weighted gene association networks and construct a much larger WGAN, which includes richer biology information while still keeps high functional relevance between linked gene pairs. The new WGAN shows satisfactory performance in disease gene prediction, which suggests the reliability of our integration strategy. Compared with existing integration methods, our method takes the advantage of the inherent characteristics of the component networks and pays less attention to the biology background of the data. It can make full use of existing biological networks with low computational effort.

## Introduction

In recent years, high-throughput biological experimental techniques[1, 2] have generated massive omic data sources at the molecular level, such as protein-protein interaction data[3], gene co-expression data[4], and transcriptional regulation data[5]. Arduous efforts have been dedicated to unravel the interplays between all genes in organisms by integrating these data into interaction networks [6–10]. In these networks, nodes represent genes, edges represent interactions between genes, and edge weights are evidence scores of the interactions fused from various biological data sources[11, 12]. Network-based approaches not only have provided more convenient platform for discovering more abundant interactions of genes, but also been employed to infer new disease genes based on links with known disease genes.

In previous studies, there are two main methods to integrate various biological functional data into a comprehensive network. One is subjective scoring integration method, and the other is statistical inference scoring algorithm.

The subjective scoring integration method first rationally evaluates the likelihood of functional coupling between genes by analyzing the objective information, and then scores the genes’ interaction by a mathematical function. Various kinds of information are used to judge the confidence of interactions between genes, such as the reliability of experimental techniques, number of validation studies and orthologous appearance in model organisms. In principle, interactions identified by low-throughput experiments such as *in vitro* X-ray crystallography get higher scores than those obtained from high-throughput experiments such as affinity based technologies. Schaefer *et al* generated a scored human PPI network HIPPIE (Human Integrated Protein-Protein Interaction rEference) from multiple sources, and developed an expertly curated scoring scheme that takes into account three types of information: superiority of experimental techniques to identify the PPI, numbers of studies that have found the PPI, and orthologs of the interacting proteins that have been found experimentally to interact in other model organisms[13]. The interactions are mainly retrieved from several public databases, such as BioGrid[14], IntAct[15], MINT[16], DIP[17], and BIND[18].

Statistical inference scoring method rescored the possibility of associations between genes based on prior information, which provides some basic evidence for the interaction of gene pairs[19–22]. We classify this statistical scoring method into two categories. One is based on probability likelihood ratio scoring, and the other is based on Bayesian method. Lee *et al* used a log likelihood scoring (*LLS*) scheme to construct a genome-scale functional network of human genes called HumanNet, which incorporated diverse data sources, such as gene expression, protein interaction, genetic interaction, sequence, literature, and comparative genomics data. This network includes data directly collected from human genes, as well as those from orthologous genes of yeast, worm, and fly [23]. They utilized HumanNet to predict disease genes and the results showed a favorable performance. Mering *et al* generated a human gene association network STRING using a naive Bayesian model, which integrated known associations collected from many public databases and predicted associations via gene neighborhood, gene fusion, gene co-occurrence, and gene co-expression[20, 24, 25]. They scored each gene pair based on several evidences, such as the co-membership in KEGG pathways, which support their functional coupling. Additionally, Alexeyenko *et al* mixed information derived from proteomics and genomics pipelines and generated a human gene association network FunCoup by an optimized Bayesian framework[19]. Then, they used the network FunCoup to construct a protein-protein interaction network associated with the disease Alzheimer[26]. STRING and FunCoup are scored by similar method which integrated various evidences supporting the associations between genes.

The existing human genome gene association networks, such as HumanNet, STRING and FunCoup, have successful applications in biological research and disease gene prediction [27–29]. However, there is a huge difference between these networks. We find that they share more than 80% common genes, but the common edges are very limited (the proportion of common edges to total number is less than 10%). Therefore, it is necessary to integrate the existing networks to obtain a weighted gene association network, which contains more abundant information by making best use of the information of previous networks. Such work could be valuable to understand cellular processes and study the pathology of complex diseases[30, 31].

In this paper, we propose an algorithm based on information entropy [32–36] to integrate multiple weighted gene association networks (WGANs). Using this algorithm, we integrate four existing human gene association networks to construct a much larger network. To verify the reliability of this integrated network, we use it as background network to predict disease genes and compare its performance with the original networks.

## Materials and methods

### Data resources

In this paper, an integration strategy is designed to integrate different weighted gene association networks, and an example is provided to show the integration performance. In our example, four human gene association networks are constructed from different databases, and a network called GO [37–39] is used as a test network for the selection of parameters in the integration model. Detailed information of the networks is presented as follows.

1. HIPPIE: a scored human PPI network integrated from multiple sources, which used an expertly curated scoring scheme that takes into account the reliability of three types of information. The authors aimed to map many gene pairs in different public databases and give them a score calculated by analyzing the reliability of three types of information.
2. HumanNet: a genome-scale functional association network of human genes which were integrated from 21 large-scale genomics and proteomics datasets. In this network, the edge weights are structured by calculating the log likelihood scoring (*LLS*) for each pair and integrating these *LLS* to a final weight.
3. FunCoup: a genome-wide functional association network constructed from the version 3.0 of FunCoup database, which integrates large amounts of genomic data by an optimized Bayesian approach.
4. STRING: a gene association network constructed from the version 9.1 of SRING database which aims to collect and predict many types of gene-gene associations, including physical and functional interactions from diverse sources. In the network, the weight of each link represents a probabilistic confidence score.
5. GO: a human gene association network constructed from the Gene Ontology database which provides structured, controlled vocabularies and classifications that contain several domains of molecular and cellular biology. In this network, there is a link if two genes share at least three GO terms, while the number of shared terms is assigned as weight of current link.

Basic information of above networks is listed in Table 1. Since genes in these databases are presented in multiple identifiers and are obtained by distinct algorithms, we first map the identifiers into the Entrez gene codes and then normalize the weight of each edge into the area (0, 1].

**Table 1:** Basic information of the four original networks (HIPPIE, HumanNet, FunCoup and STRING) and the GO network.

Network	HIPPIE	HumanNet	FunCoup	STRING	GO
Nodes	16,514	16,243	16,626	16,213	18,386
Edges	235,184	476,399	4,044,929	3,180,982	45,449,515
Average degree	28.48	58.66	486.58	392.40	4943.93
Average clustering coefficient	0.129	0.246	0.438	0.232	0.786

### The workflow for the integration of networks

**Fig 1:**
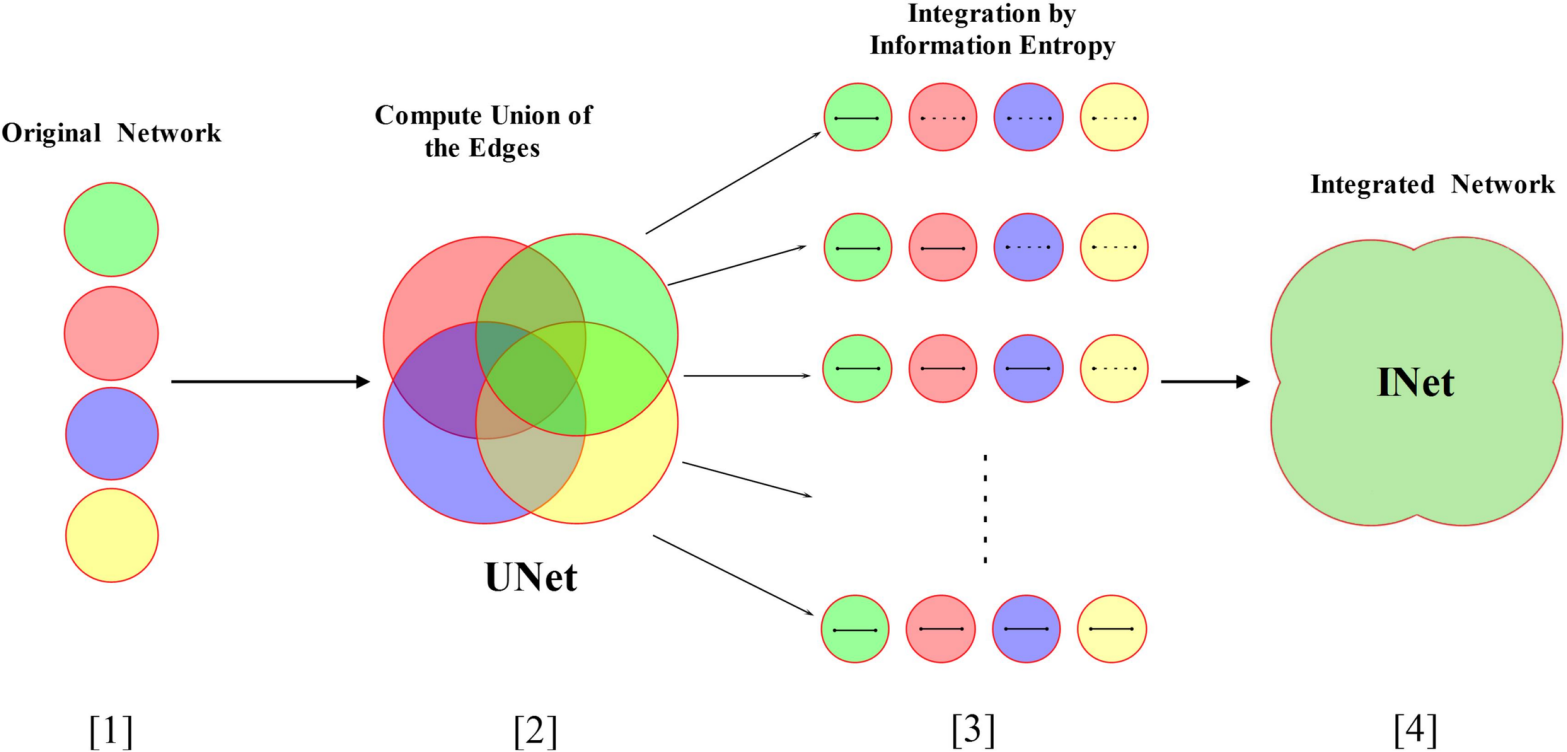
The workflow for the integration of networks. Circles with different colors denote different networks. The solid line in circle means an edge existing in the network and the dashed line means an edge absent in the network. In Step [3], each row denotes the same pair of genes which is connected in at least one of the networks.

In this section, we outline the workflow that integrates these four networks (see Fig 1) The steps are as follows:

Construct the four networks from the databases and normalize the edge weight to the area (0, 1].

1. Combine all the nodes and edges of the four networks to construct a union network denoted by UNet.
2. For each edge (*i*, *j*) in the union network UNet, rescore the edge weight by the model of information entropy.
3. Construct the integrated network (denoted as INet), which has the same nodes and edges as the union network while the edge weights are obtained in step 3.

### Integration model based on information entropy

We employ information entropy to integrate the weights of the four networks. Generally speaking, entropy is a variable used to measure the disorder degree of a system. It has specific explanations in different areas. Considering a random event, entropy describes the mean uncertain degree of the random variable. This measure has also been widely used in the field of information theory, which is known as information entropy.

For a random variable *X*, if *X* contains *n* possible instances denoted by *x*_*i*_(*i* = 1,2…*n*) whose occurrence possibility is *p*_*i*_ = *P*(*x*_*i*_), the uncertain degree of the occurrence of *X* can be defined by information entropy as follows:

$$H(X)=-\sum_{i=1}^{n}p(x_{i})*\log_{2}p(x_{i})$$ (1)

Suppose there are *m* WGANs to be integrated, which are denoted by *Net*_1_,⋯,*Net*_*m*_. We combine all edges in the *m* WGANs to obtain an edge union set E_U_ and construct a union network UNet using all the edges and nodes in the set E_U_. Then our task is to calculate a weight for each edge in the network UNet from weights of the *m* original networks. For each gene pair (*i*, *j*) in set E_U_, $W_{k}^{\left(ij\right)}$ is the edge weight of the gene pair (*i*, *j*) in the *k*th network. We further integrate the edge weight of gene pair (*i*, *j*) in *m* WGANs into a new evidence score of the integrated network as follows,

$$W^{\left(ij\right)}=\alpha_{1}^{\;(ij)}W_{1}^{\;(ij)}+\ldots +\alpha_{m}^{\;(ij)}W_{m}^{\;(ij)}$$ (2)

where $\alpha_{1}^{\;(ij)},\alpha_{2}^{\;(ij)}\ldots \alpha_{m}^{\;(ij)}$ are positive numbers which represent the integration parameters of gene pair (*i*, *j*) in corresponding networks. Larger $\alpha_{k}^{\;(ij)}$ suggests larger contribution of the corresponding network *Net*_*k*_ in the integration (*k* = 1,2,…*m*), $\alpha_{1}^{\;(ij)}+\ldots +\alpha_{m}^{\;(ij)}=1$.

To design reasonable integration parameters, we describe the average uncertain degree of an edge existing between a gene pair (*i*, *j*) by information entropy and use it to define the integration parameters. As we know, *W*^(*ij*)^ can be explained as the probability that the edge exists between gene *i* and *j* in a weighted gene association network. For the sake of convenience, we define the following random variable *Y*,

$$Y=\left\{ \begin{array}{ll} 1 & i\;\mathrm{and}\;j\;\mathrm{are}\;\mathrm{linked}\\ 0 & i\;\mathrm{and}\;j\;\mathrm{are}\;\mathrm{not}\;\mathrm{linked} \end{array}\right.$$ (3)

$$P(Y=1)=W^{\left(ij\right)},P(Y=0)=1-W^{\left(ij\right)}.$$

Therefore, the random variable *Y* describes whether there are interactions between node *i* and *j*. According to information entropy described in Eq (1), we can describe the uncertain degree of the interactions between node *i* and *j* as following:

$$H(W^{\left(ij\right)})=H(Y)=-W^{\left(ij\right)}\log_{2}W^{\left(ij\right)}-(1-W^{\left(ij\right)})\log_{2}(1-W^{\left(ij\right)})$$ (4)

In practice, one pair of genes may have interaction in some of the original networks, and have none interaction in the rest of networks. For the latter, it is mathematically meaningless when computing information entropy to depict the uncertain degree of gene pairs. Thus it is necessary to preprocess such pair of genes. In this case, we assume that there is an interaction between the gene pair (*i*, *j*) in the network and give it a rather small weight *ε*. Similarly, for a gene pair (*i*, *j*) with edge weight 1, we also change its weight as 1−*ε*. In this paper, we set *ε* = 0.001. In fact, we choose *ε* = 0.001 just because it is far smaller than each weight of the existing edges in the original networks.). In this way, each edge (*i*, *j*) in the union set EU owns *m* weights $W_{k}^{\left(ij\right)}(1\leq k\leq m,0<W_{k}^{\left(ij\right)}<1)$.

For the *k*th WGAN, the edge weight of gene pair (*i*, *j*) is $W_{k}^{\left(ij\right)}$, thus the entropy that an edge exists between the gene pair (*i*, *j*) is

$$H(W_{k}^{\;(ij)})=-W_{k}^{\;(ij)}\log_{2}W_{k}^{\;(ij)}-(1-W_{k}^{\;(ij)})\log_{2}(1-W_{k}^{\;(ij)})$$

The larger the entropy $H(W_{k}^{\;(ij)})$, the greater the uncertain degree of existing edge between the gene pair (*i*, *j*). Therefore, the entropy $H(W_{k}^{\;(ij)})$ can be used to design the integration parameters $\alpha_{k}^{\;(ij)}(1\leq k\leq m)$ in Eq (2). It is reasonable to give a relatively small value to $\alpha_{k}^{\;(ij)}$ if the entropy $H(W_{k}^{\;(ij)})$ is large. We define a function which decreases with the increasing of $H(W_{k}^{\;(ij)})$ as follows,

$$C_{k}^{\left(ij\right)}=C(W_{k}^{\;(ij)})=1-e^{-\frac{1}{{\left[H(W_{k}^{\;(ij)})\right]}^{\theta }}}$$ (5)

where *θ* > 0 is an adjustment parameter, which can be properly selected by training with real data. Our function is specifically designed to restrict the integration parameter $C_{k}^{\;(ij)}$ in the area (0, 1).Then we design the integration parameters $\alpha_{k}^{\;(ij)}$ by normalizing $C_{k}^{\;(ij)}$ as follows:

$$\alpha_{k}^{\;(ij)}=\frac{C_{k}^{\;(ij)}}{\sum_{s=1}^{m}C_{s}^{\left(ij\right)}},k=1,\ldots ,m$$ (6)

Lastly, for any edge (*i*, *j*) in the union set E_U_, by integrating the edge weights $W_{k}^{\left(ij\right)}(1\leq k\leq m)$ given in the *m* networks, the new edge weight *W*^(*ij*)^ of the gene pair (*i*, *j*) in the integrated network is derived according to Eq (2).

**Fig 2:**
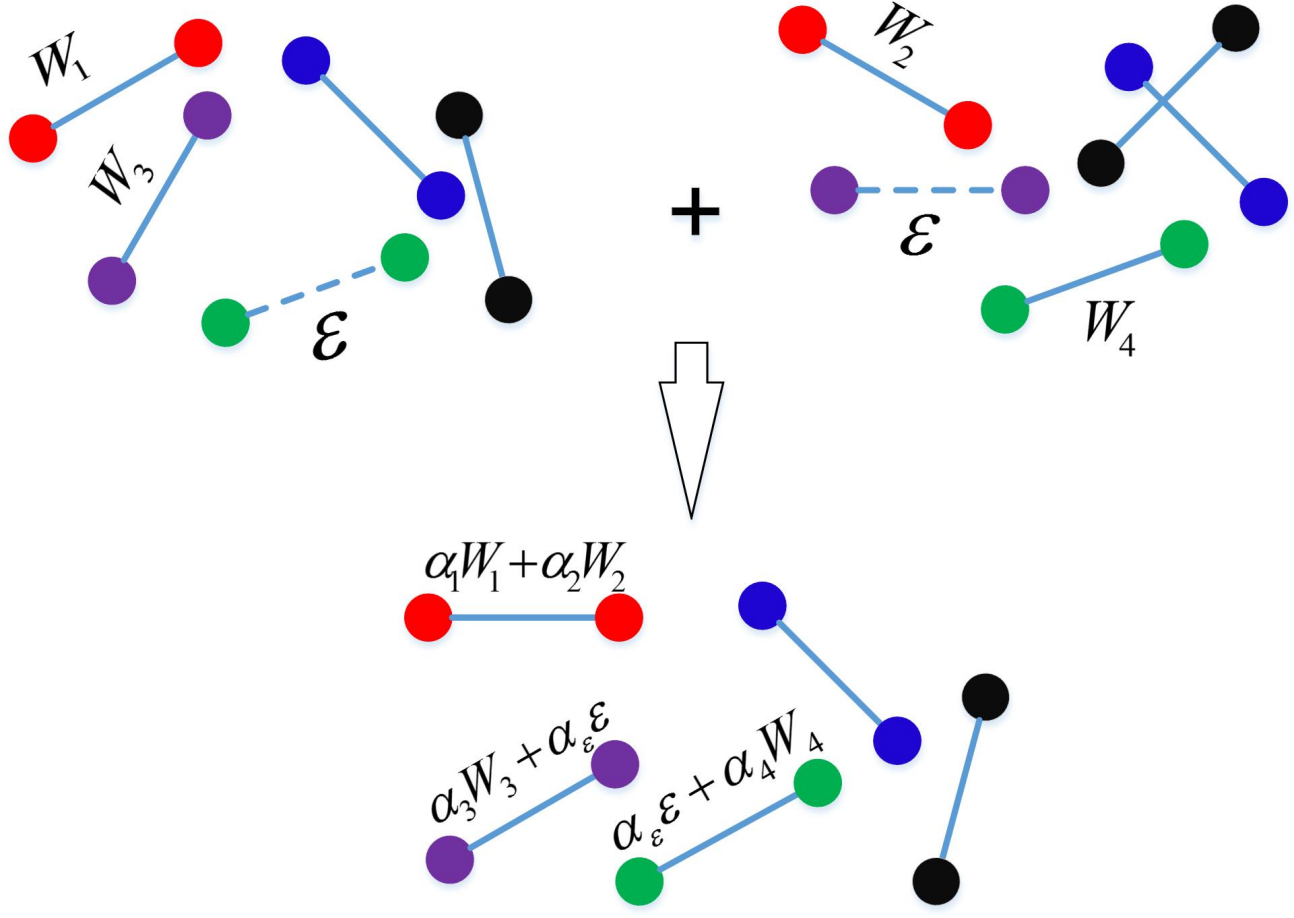
A simple example of integrating two networks.

To sum up, our algorithm mainly includes the computation of information entropy for each edge in the original networks and edge weights in the integration process. Firstly, computations of information entropy for each edge in the UNet are polynomial whose computational complexity is *CCE*<*O*(*e*), where *e* is the number of edges in the four original networks. Secondly, computations of edge weights in the integration process are polynomial. Thus total computational complexity is *CC* = *CCW* × *ES* × *CCE*. Here, *CCW* is the computational complexity of edge weights, *ES* is the number of edges in the four original networks, *CCE* is the computational complexity of information entropy. Therefore this algorithm is polynomial. Fig 2 shows a simple example of integrating two networks.

### The selection of the adjustment parameter in the integration model

In this section, we select the adjustment parameter *θ* in Eq (5) for a more accurate integration.

We construct the GO network according to the functional information of all human genes in the gene ontology database (GO), and use it as a test network to determine the adjustment parameter *θ* of the integration model. GO database is established by Gene Onotology Consortium, which describes almost all known genes in multiple species and annotates them with biological function by standard lexical items (GO term). A GO term represents a specific biological meaning the gene owns. The more GO terms two genes shared, the higher biological similarity they have. Generally, two genes can be linked in the network if they share at least one common GO term. In order to enrich our selection, we finally take gene pairs which share at least three common GO terms, and the number of shared terms is assigned as weight of the current link. Afterwards, we normalized the edge weight into the interval of (0, 1]. Finally, a normalized GO network is constructed with its edge weight proportional to the biological similarity. Since the GO database is established entirely from biological knowledge without using any computational data, this network is different from the networks we used as sources for integration. Therefore, we use it as the comparing background to adjust the parameters.

**Fig 3:**
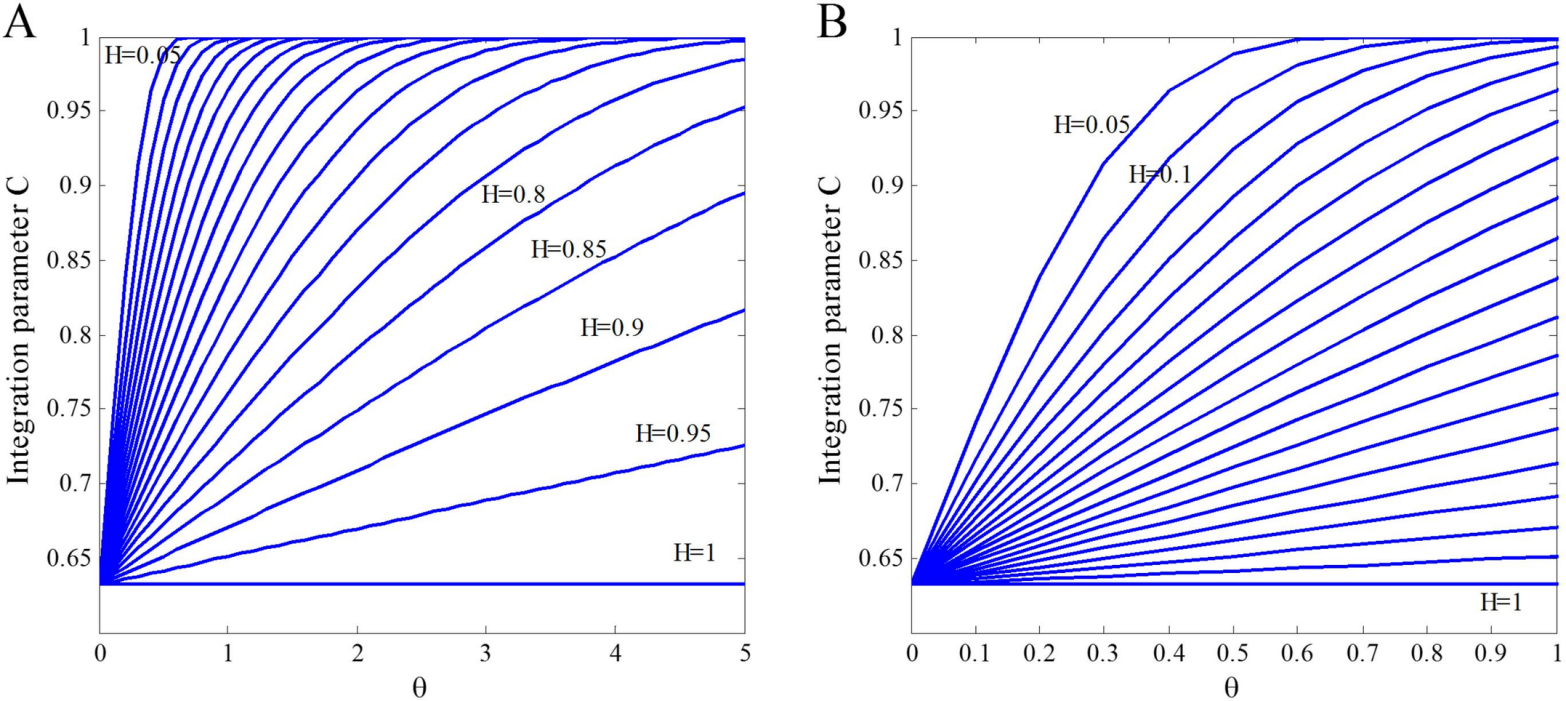
Variation trend of the integration parameter *C* with the change of the adjustment parameter *θ* and information entropy *H* based on the Eq (5). (A) *θ* changes in the area [0, 5]; (B) *θ* is limited to the area [0, 1].

*θ* is the adjustment parameter which is used to adjust the influence of information entropy of the pair (*i*, *j*) on the integration parameter. In order to get a reasonable adjusting interval of *θ*, we need to observe that how the integration parameter *C* changes with *θ* and *H* in Eq (5). In Fig 3A we set *θ* in the area [0, 5] and plot the variation trend of *C* with the change of *θ* and *H*. It shows that, for small values of *H* (such as *H* = 0.05, 0.1), when the value of *θ* is larger than 1, the value of *C* has no significant difference. Thus in Fig 3B we limit *θ* in the area [0, 1]. We can see that, for different values of *H*, the area [0.2, 0.6] of *θ* makes *C* a relatively significant difference. Therefore, we set the area for adjusting the parameter *θ* as [0.2, 0.6].

Finally, *θ* can be determined by solving the following optimization problem:

$$\underset{\theta }{\min}f(\theta )=\underset{\theta }{\min}{\sum \left(W_{\theta }-W_{GONet}\right)}^{2}\;\theta \in [0.2,0.6]$$ (7)

where *W*_*θ*_ represents the weights of the common edges with GO network in the integrated network, *W*_*GONet*_ is the weight of the corresponding edge in the GO network.

### Network-based disease gene prediction

Weighted gene association networks provide a new platform for the study of complex diseases in the context of molecular networks [40–45]. As pointed out in previous works, the functionally related genes tend to cluster in the network and result in same or similar disease. Thus it is worthwhile to infer potential disease genes by network modeling. Based on such observation, we take genes known to be associated with a particular disease as network 'seeds', and then rank the candidate genes according to their proximity with the seeds by neighborhood weighing rule[46]. Specifically, for a given disease, each gene *i* in the network is prioritized according to the sum of the weights of its links to the known disease (seed) genes:

$$S_{i}=\sum_{j\in seed}W_{ij}$$ (8)

where *W*_*ij*_ is the weight of the link between genes *i* and *j*. If gene *i* has no links with any seed genes, *S*_*i*_ is 0.

To test the quality of the final integrated network, we use it and the four original networks as background network respectively. Disease gene prediction is conducted in each background network respectively and the performances are compared. We conduct two kinds of experiments as follows.

#### Evaluating the power of the networks in disease gene prediction using leave-one-out cross validation

We extract 609 distinct disease genes from the Online Mendelian Inheritance in Man (OMIM) database. These genes are assembled into 113 seed gene sets corresponding to 113 disorders of human disease phenotypes, in which each seed set contains at least 3 genes. Next, we evaluate the integrated network by leave-one-out cross validation[47, 48] based on the neighborhood weighing rule. This evaluation treats each known gene-disease set as a test case, and assesses how well each known disease gene ranks against a background set of genes when the remaining disease genes are used as seeds. Then, all test cases are pooled together to evaluate the overall performance.

#### Evaluating the power of the networks in predicting new disease genes

DisGeNET is a discovery platform which provides open access to one of the largest collections of genes and variants associated with human diseases. We extracted 519 genes associated with 49 diseases from the DisGeNET [49] database, in which the 49 diseases are also included in the OMIM database. Then, we took genes in OMIM as seeds and genes in DisGeNET but not in OMIM as test genes to predict new disease genes using the final integrated network and four original networks as background network, respectively. All candidates (including the 519 disease genes) are ranked based on their connectivity with the seed set.

Afterwards, we extracted 24 known disease genes of obesity[50] from the OMIM database and 367 disease genes searched from the literature by Hancock *et al* [51]. We take 24 known disease genes of obesity as seeds and 367 related disease genes as test genes, and conduct disease gene prediction using the final integrated network and four original networks as background network, respectively. For each gene *i* in the background network, we first calculate *S*_*i*_ by Eq (8), and rank them in descending order.

#### Performance assessment

We evaluate the performance of the networks in disease gene prediction through following two criterions.

Accuracy: All test cases are pooled together, and the performance is evaluated by calculating the percentage of tested disease genes by varying rank cutoff in the interval [0, 100]. Consequently, the higher percentage the test disease genes, the better performance the background network in disease gene prediction.

AUC value: We plot ROC curves for prediction results and compute their AUC values. ROC (false positive rate vs true positive rate) curve is plotted by changing the rank cutoff from 1 to the number of all genes in the background network by turns. In detail, false positive rate is the fraction of non-seed genes ranked above the threshold, while true positive rate is the proportion of seed genes ranked above the threshold. AUC is the area under the ROC curve, which lies in the interval [0.5, 1]. It will be 0.5 if all disease genes are distributed at random in the rank, and larger area indicates better performance.

Both of the criterions are used to evaluate the performance of different networks in disease gene prediction. Percentage of test genes shows the accuracy in certain rank cutoff. AUC value shows the predicting accuracy which synthesizing all rank cutoffs in gene association network. The latter criterion suggests an overall performance and it is more robust than the former.

## Results and Discussion

### Comparison of the four original weighted human gene association networks

Although the four networks under study are all weighted association networks of human genes, they were built by different research teams. HIPPIE is a scored human PPI network integrated from multiple sources. Links in this network are weighed by an expertly curated scoring scheme, which takes into account three types of information including experimental techniques, evidence numbers, and orthologs. Networks HumanNet, FunCoup and STRING come mainly from varieties of databases constructed by fusing physical interaction data and functional association data by log likelihood scoring methods or naive Bayesian framework. The number of nodes in the four networks is almost the same, but the number of edges in each network is quite different. For example, as shown in Table 1, Networks HIPPIE and HuamnNet have rather fewer edges than networks FunCoup and STRING.

**Fig 4:**
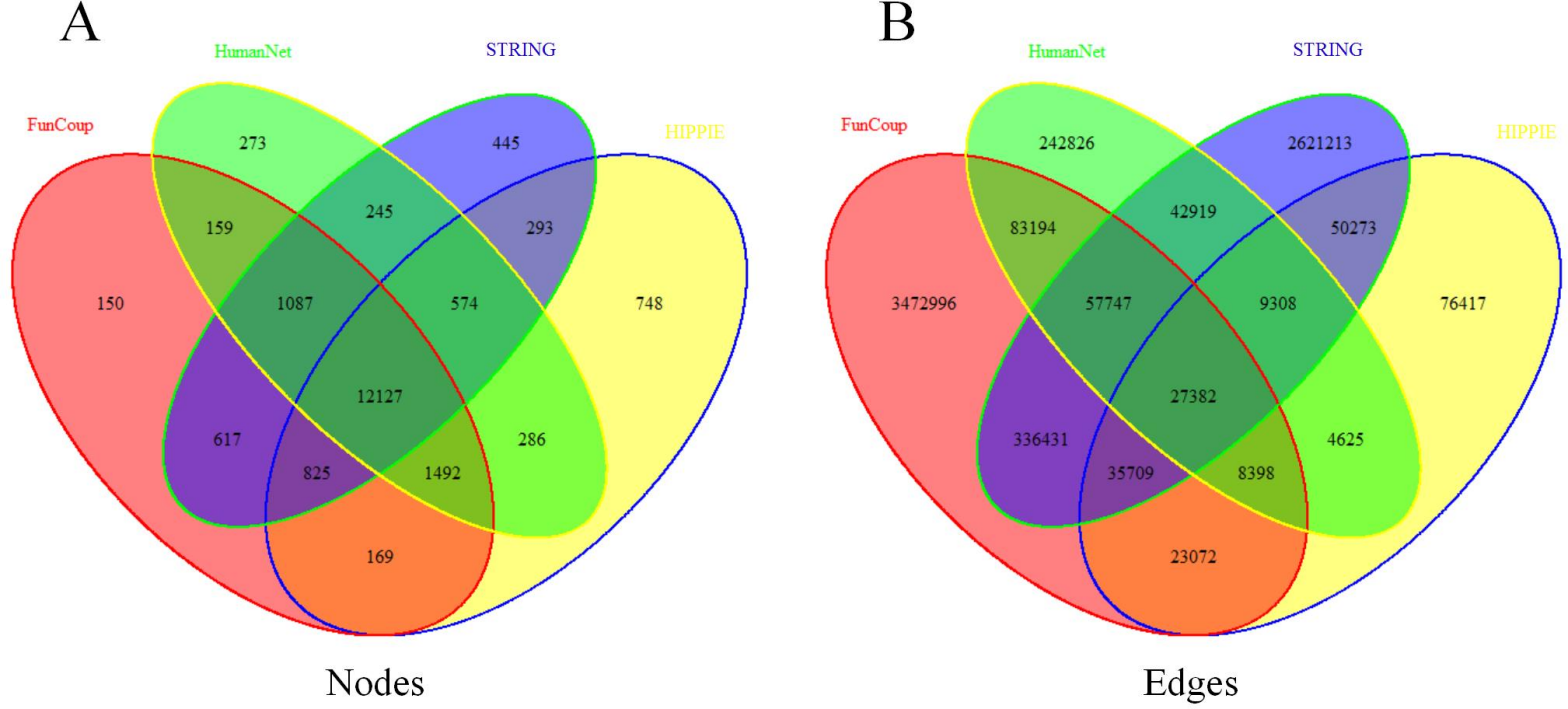
The number of overlapped nodes and edges of the four original networks under study. (A)Common nodes of the four original networks (HIPPIE, HumanNet, FunCoup and STRING). (B)Common edges of the four original networks.

**Fig 5:**
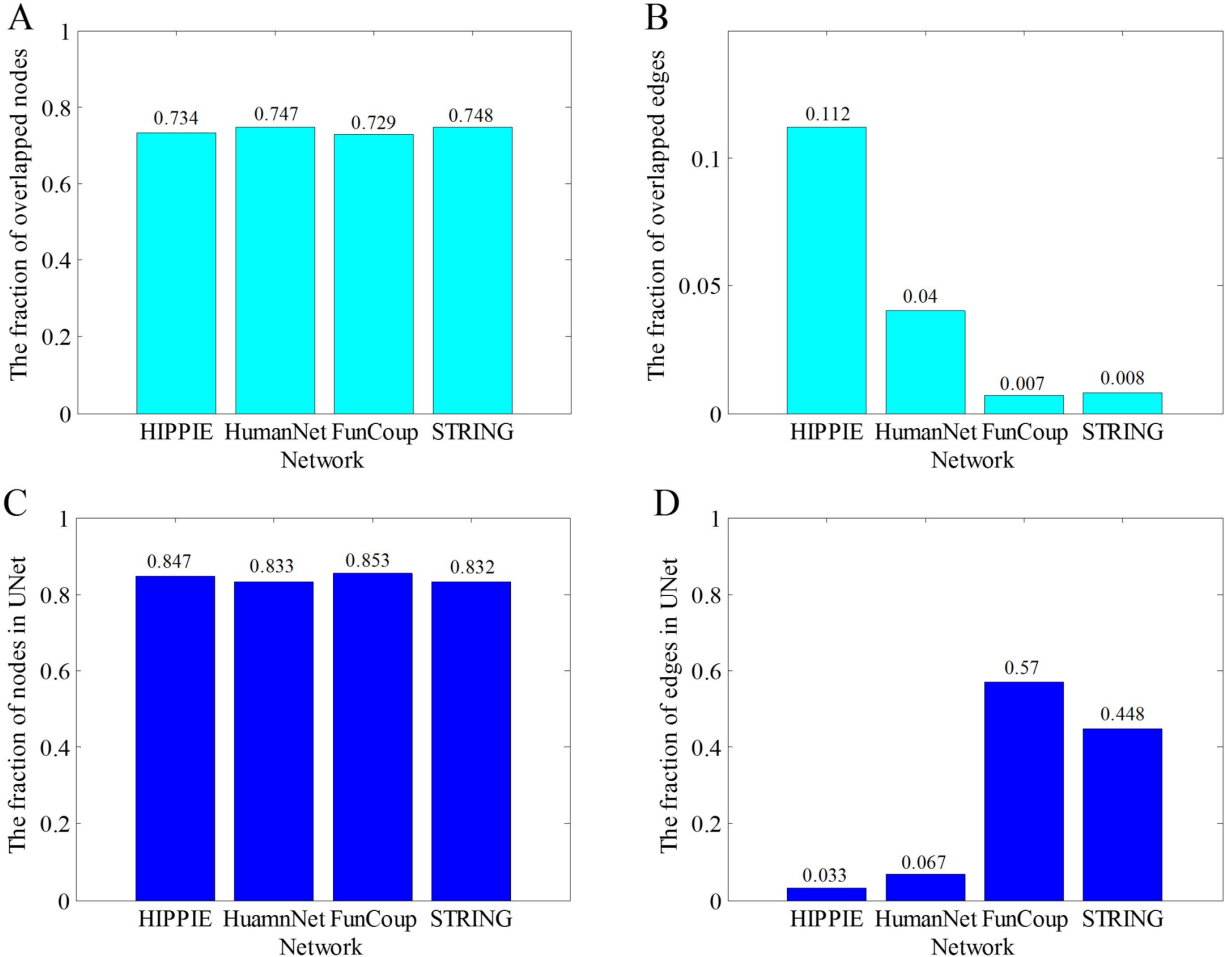
The comparison of nodes and edges in the four original networks and the union network UNet. (A) The fraction of overlapped nodes of the four original networks in their own network. (B) The fraction of overlapped edges of the four original networks in their own network. (C) The fraction of nodes of the four original networks in the union network UNet. (D) The fraction of edges of the four original networks in the union network UNet.

Fig 4 shows the information of overlapped nodes and edges between the four original weighted human gene association networks. It can be seen that they share 12,127 common gene nodes and 27,382 edges. As listed in Table 2, over 70% nodes of the four networks are common (Fig 5A). However, the numbers of common edges are rather few (Fig 5B). For example, only 0.65% edges of FunCoup appear in the other 3 networks. That is, the proportion of overlapped nodes is far larger than that of the overlapped edges.

**Table 2:** The common nodes and edges information of four networks.

Network	HIPPIE	HumanNet	FunCoup	STRING
Proportion of common nodes occupied in networks	73.43%	74.66%	72.94%	74.80%
Proportion of common edges occupied in networks	11.24%	4.05%	0.65%	0.83%

In Fig 5C and 5D, we present the fraction of nodes and edges of the four networks in the union network UNet. Each of the four networks takes over eighty percentage of nodes in the union network, while edges of each network only take very small fraction of the union, which is proportional to the numbers of edges of these networks.

The comparisons suggest that it is necessary to make full use of these networks so as to create a network that covers more information about interplays between genes.

### Construction of an integrated human gene association network by data integration

**Fig 6:**
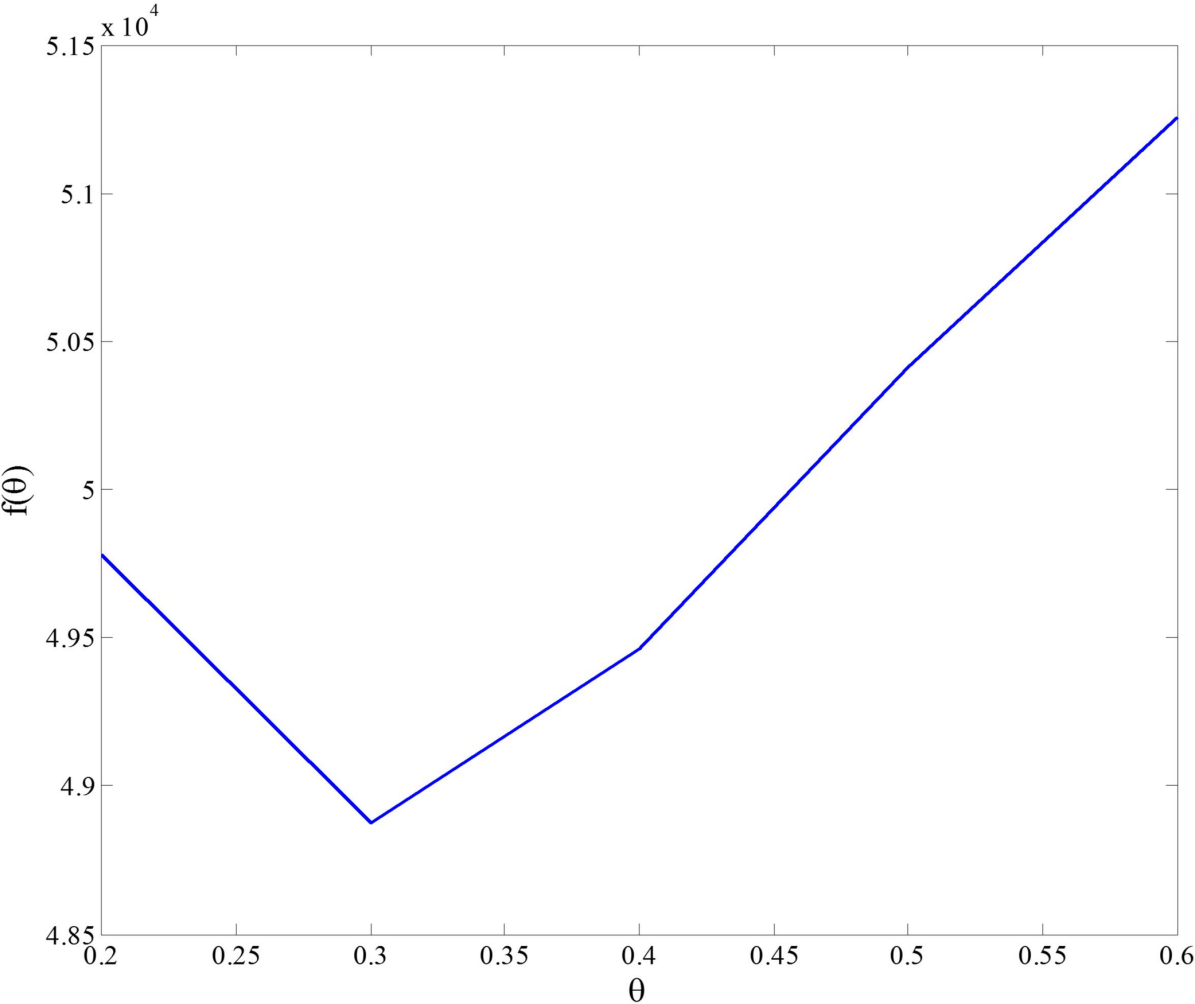
Training process to determine adjustment parameter *θ*.

Now we integrate the four weighted human gene association networks, HIPPIE, HumanNet, FunCoup and STRING according to the proposed integration model. We first combine all nodes and edges in these four networks to build the union network UNet which consists of 19,490 nodes and 7,092,510 links. Then we calculate the weight of each edge in this union network by Eq (2), in which the integration parameters are obtained by Eqs (4–6). In order to choose a proper adjustment parameter *θ* in Eq (5), we take the GO network as the training network to solve the optimization problem (7). The training result is given in Fig 6.

In Fig 6, *f*(*θ*) reaches minimum when *θ* is 0.3. Thus the adjustment parameter *θ* in Eq (5) is selected as 0.3. Then we get the weight of every edge in the union network. In this way, the integrated network (INet), a weighted network based on the union network, is obtained. See S1 File for data of this network and S2 File for the Matlab code of the integration algorithm.

**Fig 7:**
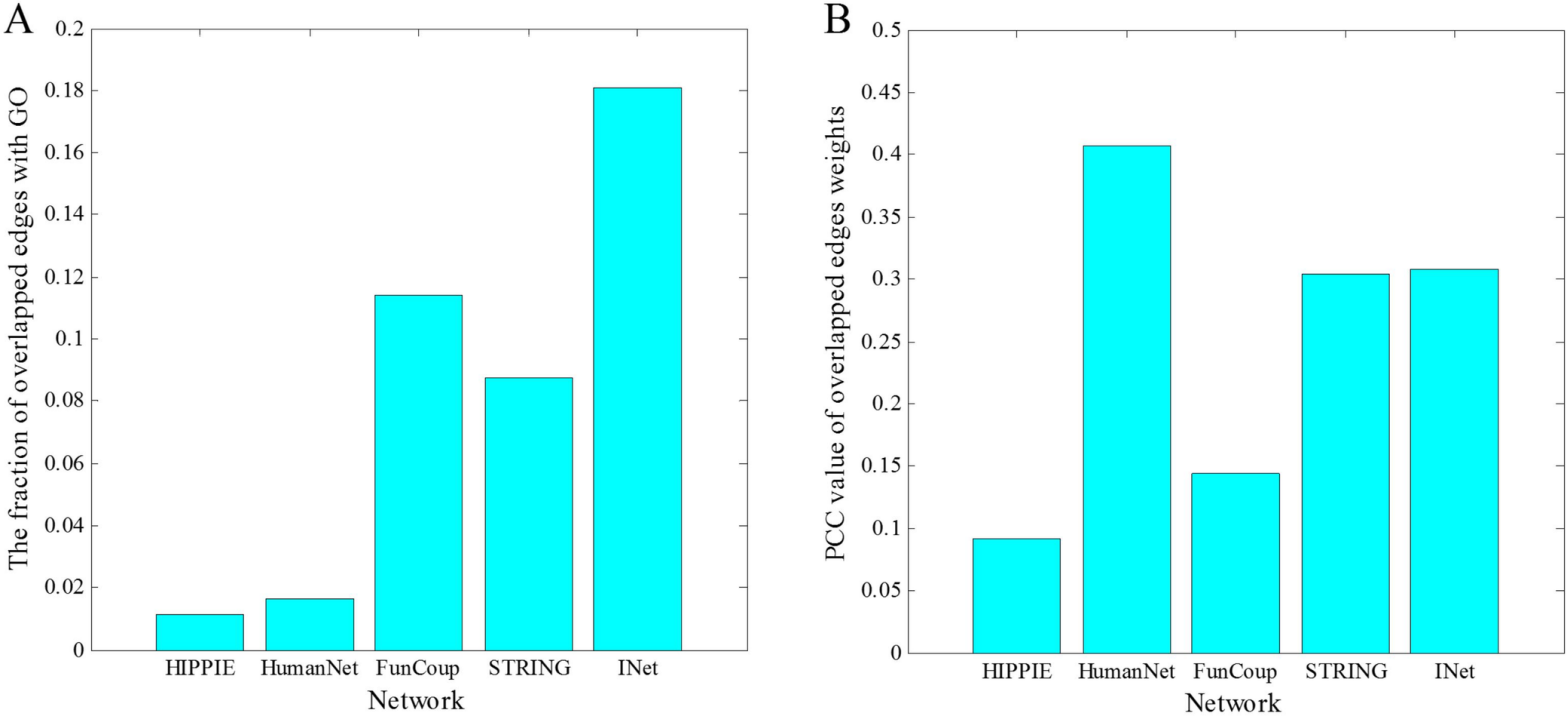
Comparisons of overlapped edges and weights information of the networks with that of the GO network. (A)The fraction of overlapped edges of the INet network and the four original networks with the GO network. (B) The Pearson correlation coefficient between the vectors for the overlapped edge weights of the network and the GO network.

Now we verify the reliability of our information entropy method for determining the edge weights. For the network INet and each of the four original networks, we first identify its overlapped edges with the GO network. Then, restricting to these overlapped edges, we calculate the Pearson correlation coefficient (PCC) between the vectors for the edge weights of the network and the GO network. All the Pearson correlation coefficients are larger than zero and all the p-values are much smaller than 0.05, implying the statistically significant positive linear correlation between the weights in all the five cases. In Fig 7A we show the fraction of overlapped edges of the INet network and the four original networks with the GO network. Since INet includes all edges of the four original networks, it has the largest fraction of overlapped edges with the GO network. Fig 7B shows that the weight of network INet has a relatively higher positive correlation with that of the GO network than most networks (higher than HIPPIE, STRING and FunCoup, but lower than HumanNet), suggesting a high functional consistency of the weight determined by our model.

The optimal *θ* is 0.3 and corresponding Pearson correlation coefficient between edges in INet and GO is 0.3075. To test the sensitivity of the parameter, we respectively set *θ* as 0.1 and 0.4 and calculated the corresponding Pearson correlation coefficient value. The results are 0.3062 and 0.3066 respectively. This result suggests that functional correlation of gene pairs in the INet is robust with the change of the parameter.

In summary, on the one hand, the integrated network provides a much larger network which includes more interaction information between genes. On the other hand, the higher correlation of its weights with those of GO implies a high functional consistency between the connected genes.

### Assessment of the integrated network in disease gene prediction

It has been known that genes associated with same disease phenotype tend to be functional related and are clustered together in gene association networks. Thus network-based methods are widely used in disease gene prediction, in which a gene association network is usually used as a background network. Here we assess the quality of the networks under study by using them as background networks for disease gene prediction.

#### Assessment by leave-one-out validation

**Fig 8:**
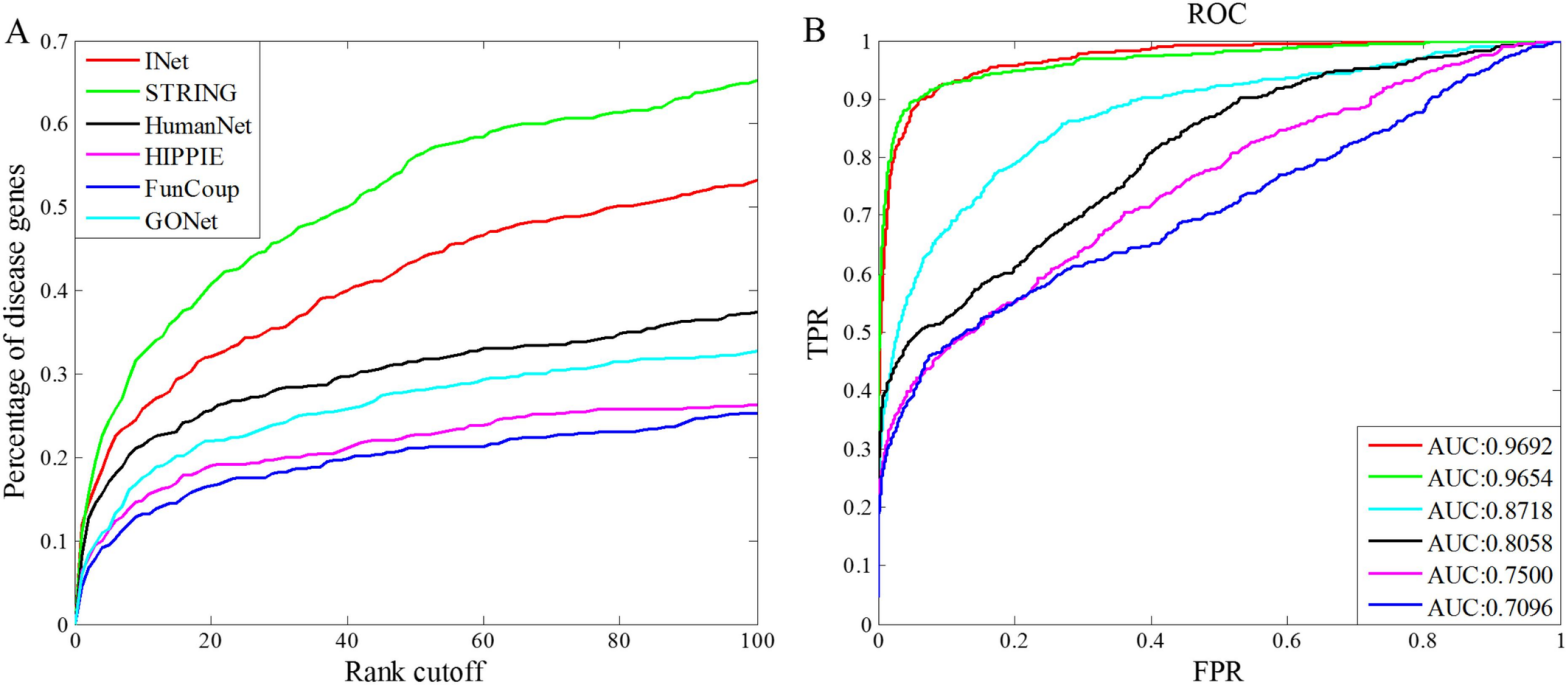
Performance comparison of disease gene prediction based on the integrated network INet, the four original networks and GO network. (A)Percentage of the test genes ranked within top 100. (B) ROC curves and AUC values for the prediction results.

We extract 609 disease genes from OMIM database, and assemble them into 113 seed gene sets corresponding to 113 disorders of human disease phenotypes. For each disease, we first extract one disease gene as a test gene. Then, we use the remaining genes as seed genes to predict the test gene. Afterwards, we get the score of each gene in the network based on the neighborhood weighing rule in Eq (8) and rank them in descending order. Last, we pool all test cases together and calculate the percentage of tested disease genes above various rank cutoffs. Besides, we plotted the ROC curve and computed the AUC value for the prediction results based on each background network. In Fig 8, we show performance comparison and ROC curves of disease gene prediction based on the integrated network INet, the four original networks and the GO network. From Fig 8A we see that INet has a much higher precision of disease gene prediction than that of HumanNet, GONet, HIPPIE and FunCoup, but poorer precision than STRING in the top 100 ranks. In Fig 8B, it is observed that the integrated network INet has the highest AUC value compared with the four original networks and the GONet. In this case, the integrated network has the highest AUC value, but it performs not better than STRING in precision of the top 100 ranks. Comparing to the precision in the top 100 ranks, AUC value shows the precision on the overall ranks. Therefore, in this leave-one-out validation, the integrated network does well in overall performance than other networks.

**Fig 9:**
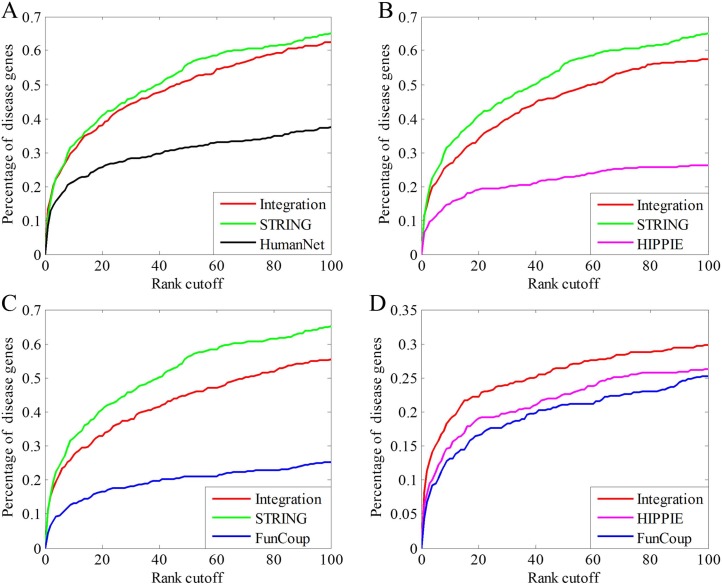
Performance comparisons of different networks in disease gene prediction by leave-one-out cross validation. (A) The integrated network is constructed from String and HumanNet; (B) The integrated network is constructed from STRING and HIPPIE. (C) The integrated network is constructed from STRING and FunCoup. (D)The integrated network is constructed from HIPPIE and FunCoup.

Considering that three of the original networks (HumanNet, HIPPIE and FunCoup) have much poorer performance than STRING, we guess that the poorer performance of INet than STRING in the top 100 precision is probably caused by them. To verify our inference, we integrate STRING with one of these three networks, respectively. In Fig 9A–9C we compare the performance of the integrated network with the component networks. In all the three cases, the performance of the integrated network is between that of the better and the poorer original networks and much closer to that of STRING. These results verify our conjecture and indicate that the performance of the integrated network could be weakened by networks which have much poorer performance. We also integrate the two networks that have the poorest performance, HIPPIE and FunCoup. As shown in Fig 9D, the integrated network exhibits better prediction precision than both of the original networks, implying that the integration has the potential to reach a “one plus one makes more than two” effect.

#### Assessment by predicting new disease genes

**Fig 10:**
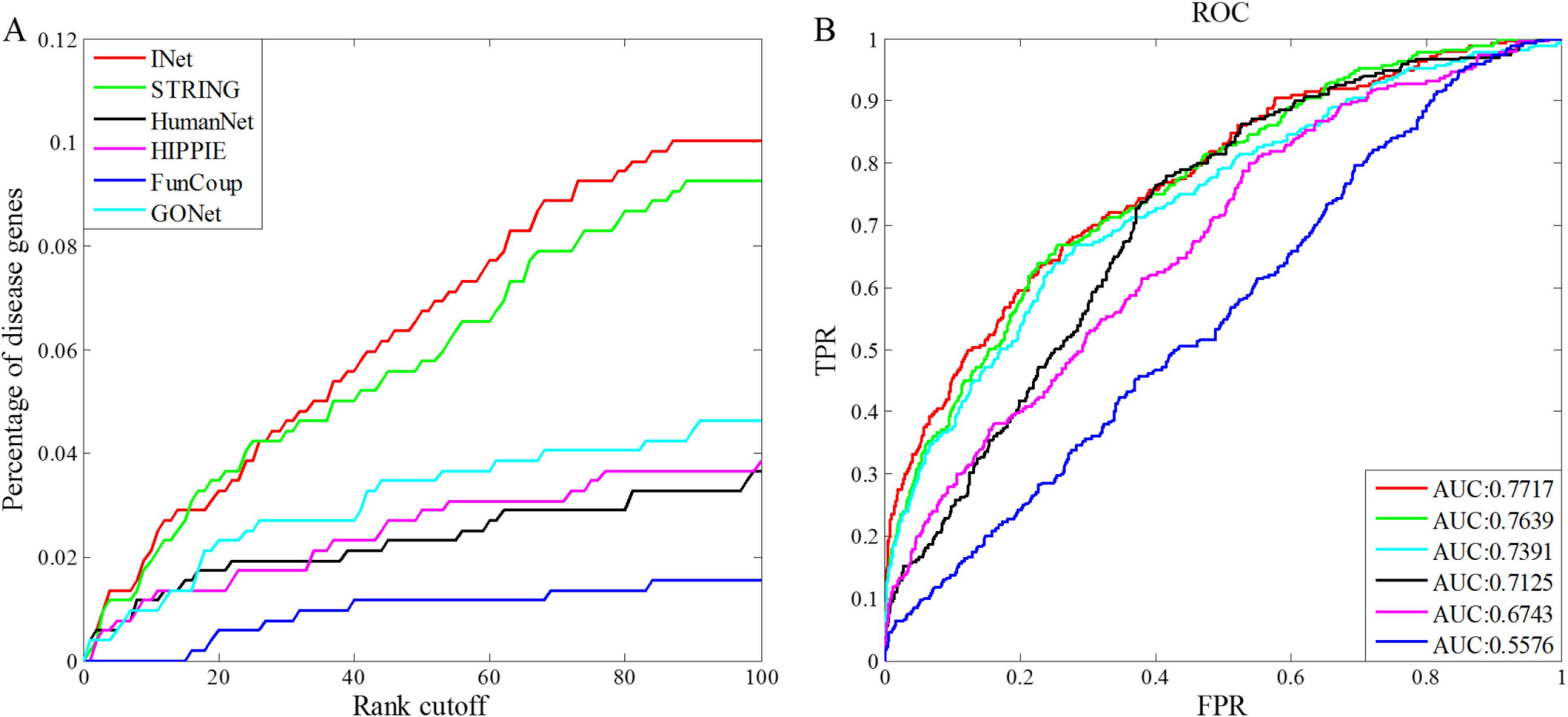
Performance comparisons for the power of different networks in predicting new disease genes. (A)Percentage of the test genes ranked within top 100. (B) ROC curves and AUC values for the prediction results.

We extracted 519 genes associated with 49 diseases from the DisGeNET database, in which the 49 diseases are also included in the OMIM database. Then, we took genes in OMIM as seeds and genes in DisGeNET as test genes to conduct disease gene prediction based on the INet and the four original networks as background network, respectively. Note that, there are no overlapped genes in seeds and test genes. As Fig 10A and 10B indicates, the integrated network not only presents the best prediction accuracy for the top 100 genes, but also has the highest AUC value than other networks. On the whole, the results of this case suggest that the integrated network could have a preferable performance than the original networks in predicting new disease genes.

### Prediction of obesity associated genes using the integrated network

To further test the reliability of the integrated network in the study of complex diseases, we mimic the search for new disease genes for the polygenic disease obesity. We use the 24 disease genes of obesity included in OMIM database as known disease genes to explore other new disease genes. We treat the 367 obesity associated genes collected from literature as unknown disease genes. In this case, the 24 genes from OMIM and the 367 genes from literature are seed and test genes, respectively. The integrated network INet, the four original networks (HIPPIE, HumanNet, FunCoup, STRING) and the GO network are background networks. We score all genes in the background network by Eq (8) and rank their scores decreasingly. Then we calculate the percentage of test genes under different rank cutoffs based on the results obtained from these networks. Also, ROC curves are plotted to compare the performance of these networks.

**Fig 11:**
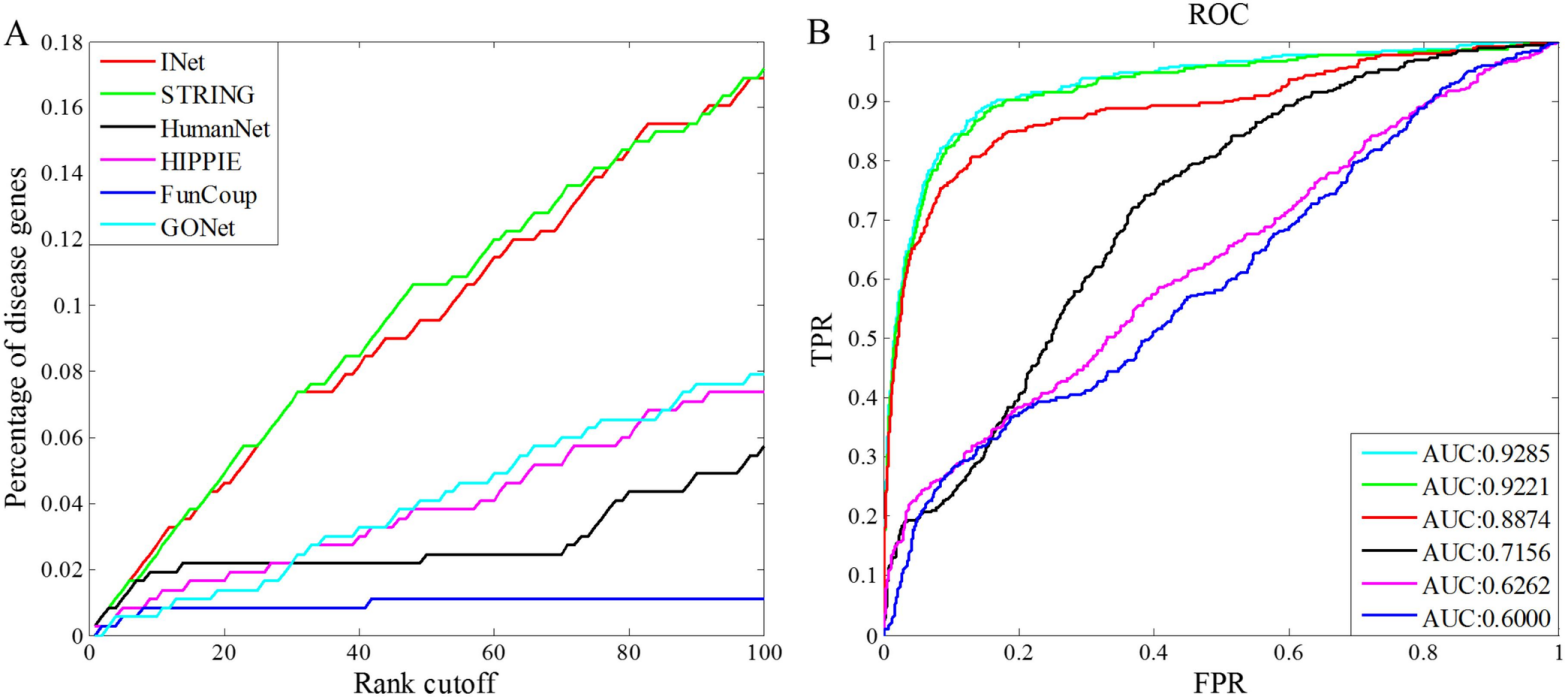
Performance comparison of obesity disease gene prediction based on different networks. (A) Percentage of the test genes ranked within top 100. (B) ROC curves and AUC values for the prediction of test genes.

Fig 11A presents the comparison between the results by INet, the four original networks and the GO network in the top 100 ranks, respectively. As shown in Fig 11A, INet and STRING exhibit the best performance among the six networks in comparison. When focusing on the top 20 ranks, INet performs more outstanding than STRING. Specifically, the top 12 genes predicted by INet are all correct, i.e., they are all included in the test set. This result further verifies good performance of INet in predicting new disease genes. Fig 11B presents the ROC curves of these networks in predicting obesity disease genes. It indicates that the GO network has higher AUC value than the other networks, although its performance is not good enough in top 100 ranks. In this case, AUC value of the integrated network is lower than GONet and STRING.

## Conclusion

In this paper, we propose a novel method based on information entropy for the integration of weighted gene association networks. We use this method to construct a weighted human gene association network from four existing networks, STRING, FunCoup, HumanNet and HIPPIE. The constructed network (named INet) includes all nodes and edges from the four original networks, while its edge weights are calculated by our information entropy algorithm using the weights in the four original networks. Edge weights of the network INet show quite high positive correlation with that of the GO network. Thus the edge weights determined by our algorithm are highly correlated with functional consistency between corresponding gene pairs. In addition, this integrated network exhibits satisfactory performance in disease gene prediction, which indicates its reliability and application significance. In summary, the network constructed from the proposed integration method includes more abundant biological information, which plays a satisfactory effect in predicting disease genes compared with previous methods. This method is insightful for the information integration of multiple weighted genomic scale gene association networks.

## Supporting information

S1 File Data of the integrated network INet.txt. (RAR)

S2 File MATLAB program for the integration of multiple weighted network. (RAR)
